# Coupled-core fiber Bragg gratings for low-cost sensing

**DOI:** 10.1038/s41598-022-05313-9

**Published:** 2022-01-24

**Authors:** Jose A. Flores-Bravo, Javier Madrigal, Joseba Zubia, Salvador Sales, Joel Villatoro

**Affiliations:** 1 Department of Communications Engineering, University of the Basque Country UPV/EHU, 48013 Bilbao, Spain; 2 Photonics Research Labs, ITEAM Research Institute, Universitat Politècnica de València, 46022 Valencia, Spain; 3 IKERBASQUE—Basque Foundation for Science, 48011 Bilbao, Spain

**Keywords:** Optical techniques, Imaging and sensing, Optics and photonics, Fibre optics and optical communications

## Abstract

Sensors based on Bragg gratings inscribed in conventional single mode fibers are expensive due to the need of a sophisticated, but low-speed, interrogation system. As an alternative to overcome this issue, in this work, it is proposed and demonstrated the use of coupled-core optical fiber Bragg gratings. It was found that the relative reflectivity from such gratings changed when the coupled-core fiber was subjected to point or periodic bending. This feature makes the interrogation of such gratings simple, fast, and cost-effective. The reflectivity changes of the gratings are attributed to the properties of the supermodes supported by the coupled-core fiber. As potential applications of the referred gratings, intensity-modulated vector bending and vibration sensing are demonstrated. We believe that the results reported here can pave the way to the development of many inexpensive sensors. Besides, coupled-core fiber Bragg gratings may expand the use of grating technology in other areas.

## Introduction

Fiber Bragg grating (FBG) sensors are increasingly accepted in more fields and sectors^1–4^ due to their multiple advantages which include, among others, compact size, high sensitivity, reliability, and immunity to electromagnetic interference. Another key advantage of such sensors is the possibility of monitoring several parameters, or the same parameter in several points, along a conventional single mode optical fiber^3,5^. The FBG technology is mature and commercially available^6^.

FBG sensors require a sophisticated readout unit to monitor the position of the Bragg wavelength (λ_B_) with high accuracy. The two most common methods to do so entail either a broadband light source and a picometer-resolution spectrometer or a tunable laser and a suitable photodetector. Both interrogation systems are bulky and costly, which makes FBG sensors expensive. As a low-cost alternative to interrogate FBG sensors, compact, integrated read-units have been proposed in the past years, see for example Refs.^7–11^. However, such miniature FBG interrogators do not compete yet in performance with the bulky ones. It is important to point out that the speed with which λ_B_ is monitored with bulk or integrated FBG interrogators is limited to a few kHz^12^.

To reduce the cost of the interrogation of FBG sensors, changes in the position of λ_B_ can be converted to intensity changes by means of edge filters^7,13–15^, interferometers^16,17^, or similar wavelength-selective devices^18^ in combination with inexpensive photodetectors. The so-called π-phase-shifted FBGs interrogated with narrow linewidth lasers have been proposed to develop intensity-modulated sensors^19–24^. The advantages in these cases include lower cost than the wavelength tracking method and much higher speeds as intensity changes can be detected from DC to several MHz^14,17,20,21,23^. However, the use of wavelength filters or π-phase-shifted FBGs has some drawbacks. One is the cost of the filter, and the other is its linear section and useful wavelength range that may be limited. Therefore, the Bragg wavelength of the grating must be properly selected. In addition, with filters or π-phase-shifted FBGs, temperature compensation may be complex.

Intensity-modulated FBG sensors are good candidates for monitoring dynamic events like impacts or vibrations^2,17^, as well as other parameters, as for example, bending or curvature^25–28^. Although in the latter case, it is important to discriminate the direction of the bending or curvature.

Based on the aforementioned, it clear that is important to investigate new methods to fabricate fiber Bragg gratings to develop functional sensors whose interrogation be less complex or expensive than the existing ones. Thus, herein, we propose the use of an optical fiber consisting of two coupled cores with FBG gratings for cost-effective optical sensing. The reflection spectrum of our gratings is a narrow peak similar to that of FBGs inscribed in conventional optical fibers. However, unlike conventional FBGs, the reflectivity of the coupled-core fiber gratings changed when such fiber was subjected to point or periodic bending (vibrations). It was also observed that the sensor provided both amplitude and direction of the bending.

The advantages of the sensors here proposed include inscription of Bragg gratings with standard procedures and an interrogation system that can be compact, simple, fast, and cost effective. No additional wavelength filters, tunable lasers or fiber devices like fan in/out are necessary. Thus, we believe that the concepts and approaches reported here may expand the use of Bragg grating technology for sensing or may represent a cost-effective alternative for sensing bending and vibrations and other parameters that can induce bending to the coupled-core optical fiber.

## Sensor fabrication and working mechanism

**Figure 1:**
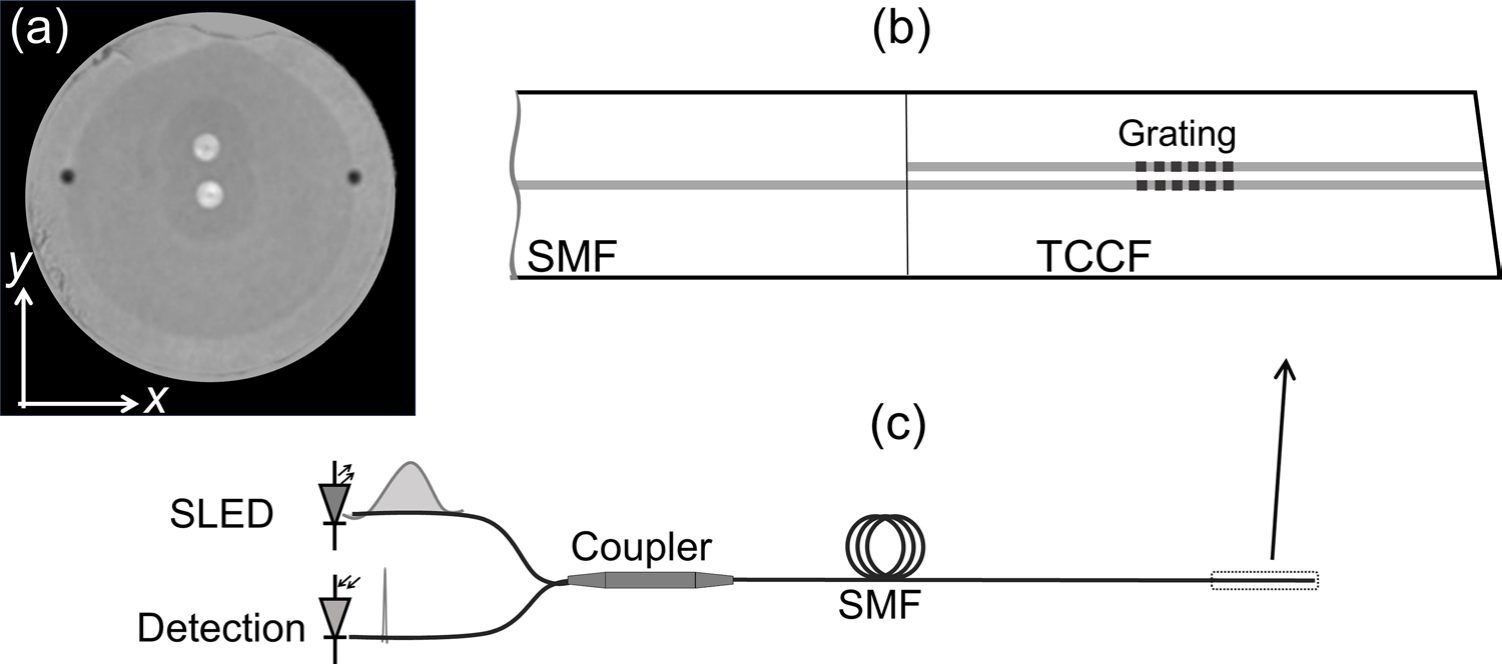
(**a**) Cross section of the twin coupled-core optical fiber (TCCF) used in the experiments. The coordinate system to orient the cores is indicated. (**b**) Schematic representation of a single mode fiber (SMF) spliced to the TCCF. (**c**) Sketch of the device interrogation; SLED is superluminescent light emitting diode. The input and reflected light are illustrated with a broad and narrow ‘spectrum’, respectively.

The cross section of the twin-core fiber that was used in our experiments is shown in Fig. 1a. The fiber was fabricated at RISE Acreo (Sweden) by drilling a void in the preform of a standard telecom fiber and inserting a second core into the void. The fiber was then drawn using conventional methods. In this manner, the fiber had two identical single-mode cores, each with a diameter of 8 μm and a numerical aperture of 0.12. One core was located in the geometrical center of the fiber and the other core was approximately at 15 μm from the central one. This short separation allows optical coupling between the cores.

A short segment of the twin-coupled-core fiber (TCCF) was fusion spliced to a conventional single mode optical fiber (SMF) that had a numerical aperture of 0.14. The splicing was carried out with a clad alignment method with a conventional fusion splicer. Under these conditions, the central core of the TCCF and the unique core of the SMF were axially aligned; see Fig. 1b. As the TCCF and the SMF have similar numerical apertures, the splice loss was low, below 0.5 dB.

To inscribe Bragg gratings in the cores of the TCCF, it was first hydrogenated at ambient temperature during 2 weeks at a constant pressure of 50 bar. A 5 mm-long grating with λ_B_ = 1552.5 nm was inscribed in the two-core fiber with the well-established phase mask technique. A frequency-doubled Argon-ion laser operating at 244 nm was used. The inscription setup is described in more detail elsewhere^29^. The two cores of the fiber had gratings with the same period, length and reflectivity (~ 35%). The position of the gratings from the SMF-TCCF junction was arbitrary as it was not critical. During the inscription of the gratings, the reflection spectrum was monitored with the setup shown schematically in Fig. 1c. Light from a superluminescent light emitting diode (SLED) with peak emission at 1550 nm was launched to the grating through a broadband optical fiber coupler. The reflected light was analyzed with a small spectrometer (IMON-512, Ibsen Photonics).

Due to the axial symmetry of the SMF-TCCF structure, see Fig. 1b, the excitation of the TCCF is carried out with the fundamental SMF mode. Such a mode excites two supermodes in the TCCF, which propagate at different velocities. As the two cores of the fiber are identical and the light is launched into the central core, thus, the normalized optical power in the central core (*P*_c_) of the TCCF at a length *L* from the SMF-TCCF junction is^30,31^:

1 $$ P_{{\text{c}}} (L,\lambda ) \, = {\text{cos}}^{{2}} (\pi \Delta n_{ef} L/\lambda ), $$

In the above equation, Δ*n*_*ef*_ is the difference between the effective refractive indices of the supermodes excited in the TCCF and λ is the wavelength of the optical source. Thus, if the transmission of an SMF-TCCF-SMF structure is measured, it will be periodic in wavelength^31,32^.

The situation when the SMF-TCCF structure where the twin-core fiber has Bragg gratings in the cores, see Fig. 1b, can be analyzed as follows. The Bragg reflection condition indicates that a specific wavelength will be reflected and that the intensity of the Bragg wavelength depends on the coupling coefficient of the forward and backward propagating supermodes. Therefore, if the TCCF is bent in the *y* direction, according to the coordinate system shown in Fig. 1a, the off-center core will experience more or less stress depending on the direction of the bending. The central core is in the neutral axis of the stress. The refractive index the off-center core is modified due to the stress caused by the bending. As a consequence, the field intensities of the supermodes, hence, the amount of light that in the cores of the fiber, will change. Therefore, changes in the reflectivity of the Bragg gratings can be expected when the TCCF is subjected to bending or vibrations.

## Results and discussion

**Figure 2:**
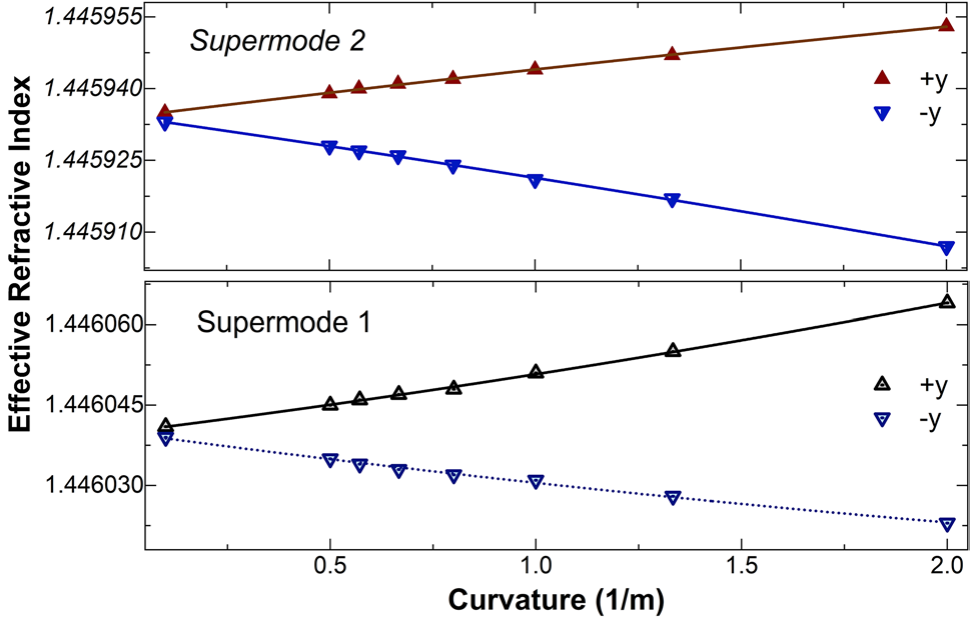
Calculated effective refractive indices (triangles) at 1552.5 nm of the two supermodes excited in the TCCF as a function of curvature. The + *y* and − *y* directions are according to the coordinate system shown in Fig. 1a. The solid and dashed lines are fitting to the calculated data.

To corroborate the aforementioned concepts, the effective refractive indices (*n*_ef_) of the supermodes excited in the TCCF were calculated at 1552.5 nm with a commercial photonic simulation software (MODE from Lumerical). The TCCF was assumed to be straight or bent with different radius of curvature in − *y* and + *y* directions, see Fig. 1a. The results of our simulations are summarized in Fig. 2. From the figure, it can be concluded that the value of *n*_ef_ of both supermodes increases when the TCCF is bent in the + *y* direction but it decreases if the fiber is bent in the − *y* direction. As it was pointed out above, the reason of this behavior is due to the asymmetry of the TCCF. The index of the off-center core of the TCCF increases or decreases depend on the direction of the bending due the stress experienced by the material the core is made of^30^.

**Figure 3:**
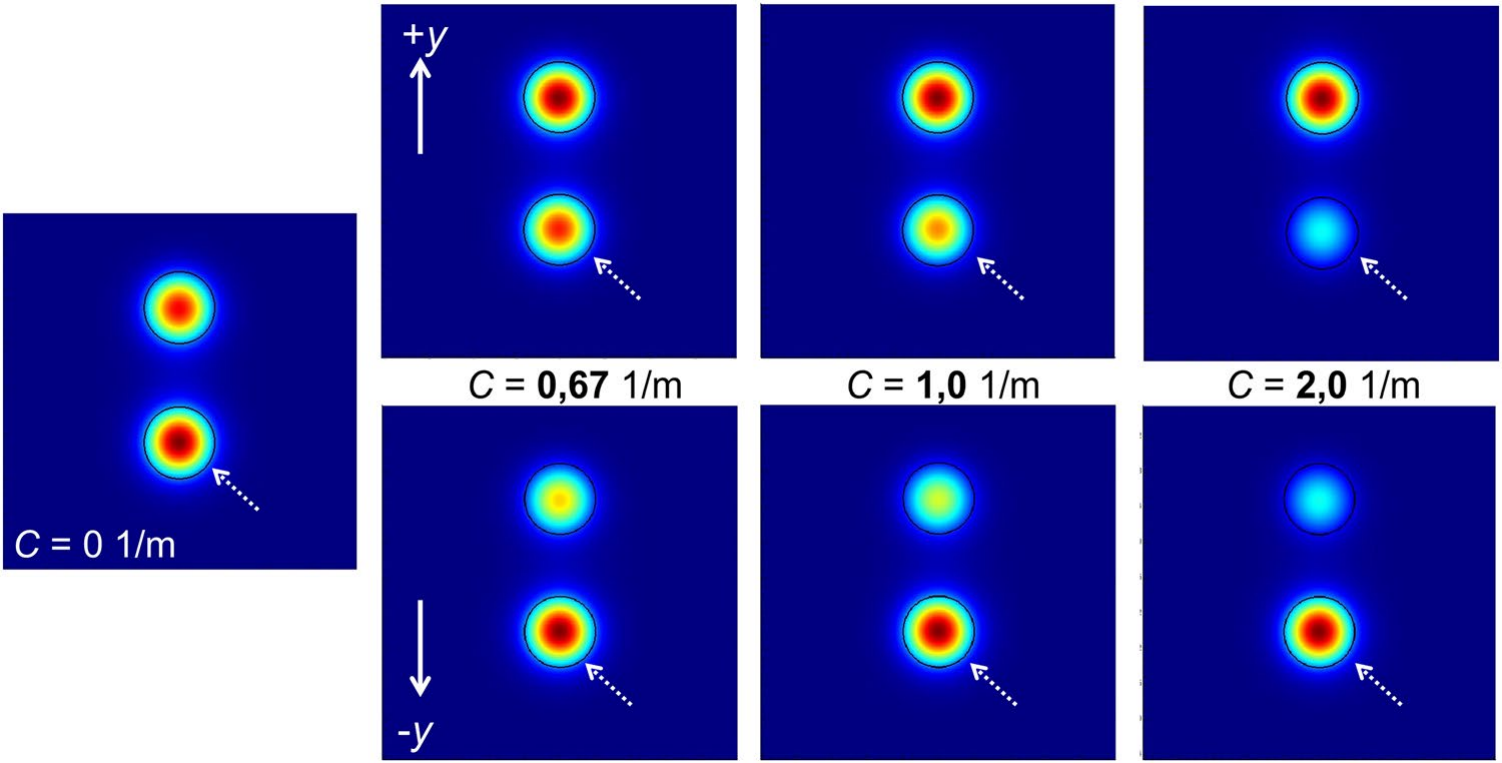
Calculated field intensities of a supermode at different values of curvature (*C*) applied to the TCCF. All the calculations were carried out at 1552.5 nm. The solid arrows indicate the direction of the bending and the dashed arrows show the central core of the TCCF. Each blue area has dimensions of 40 × 40 μm^2^.

The 2D profiles of one supermode at different values of curvature are shown in Fig. 3. The figure shows that the intensity of the supermode tends to concentrate in the off-center core of the TCCF as the value of curvature increases in the + *y* direction. This is due to the compression that such a core experiences when the fiber is bent in the + *y* direction. The opposite happens when the curvature is in the − *y* direction. In this case, the central core has more intensity than the off-center core. The distribution of intensity of the supermodes in the bent two-core fiber gives rise to changes in the reflectivity of the Bragg grating since it depends on the amount of light in the cores.

After the inscription of the gratings in the cores of the TCCF, we investigated the potential of our device for sensing bending and its direction as well as vibrations. To investigate the performance of our devices as bend sensors, the cores of the TCCF were oriented in the direction of the bending, after that, the fiber was bent in the + *y* and − *y* directions, see Fig. 1a. During the experiments, the SMF-TCCF junction was fixed and the TCCF was bent at a point located at 31 mm from the fixing point. The TCCF was angle cleaved to avoid Fresnel reflections that could affect the reflectivity measurements. A motorized translation stage was used to bend the dual-core fiber in a controlled manner. At each bending angle, the spectrum of the grating was collected with the measuring setup shown schematically in Fig. 1c.

**Figure 4:**
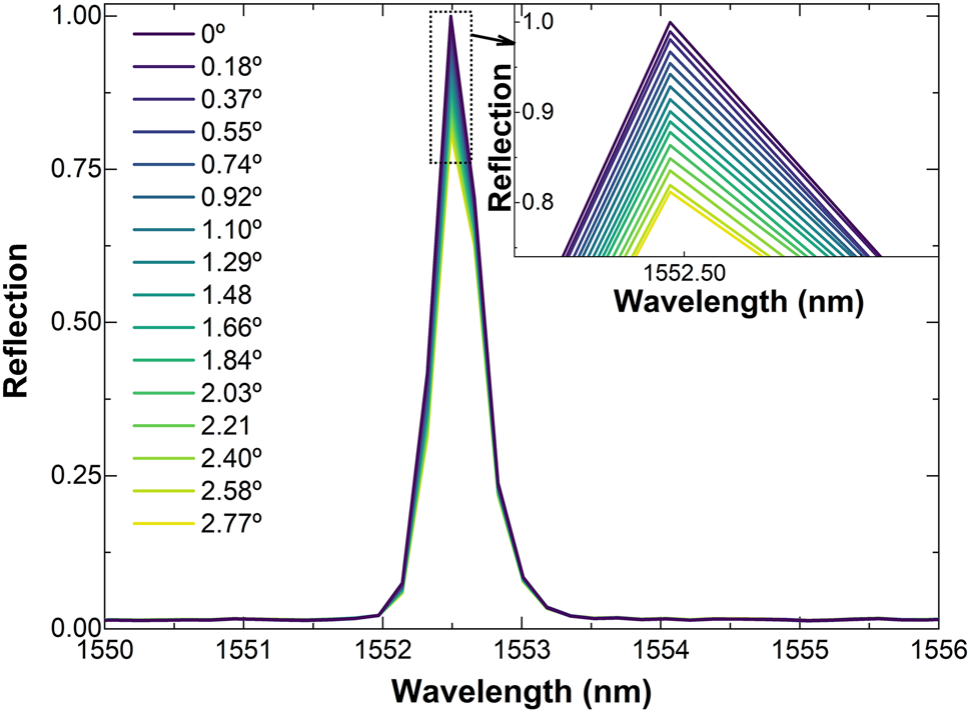
Reflection spectra for a 5-mm long Bragg grating inscribed in the TCCF at different bending angles in the − *y* direction, according to the coordinate system shown in Fig. 1a. The inset graph is a zoom in of the reflection indicated with a dotted square.

**Figure 5:**
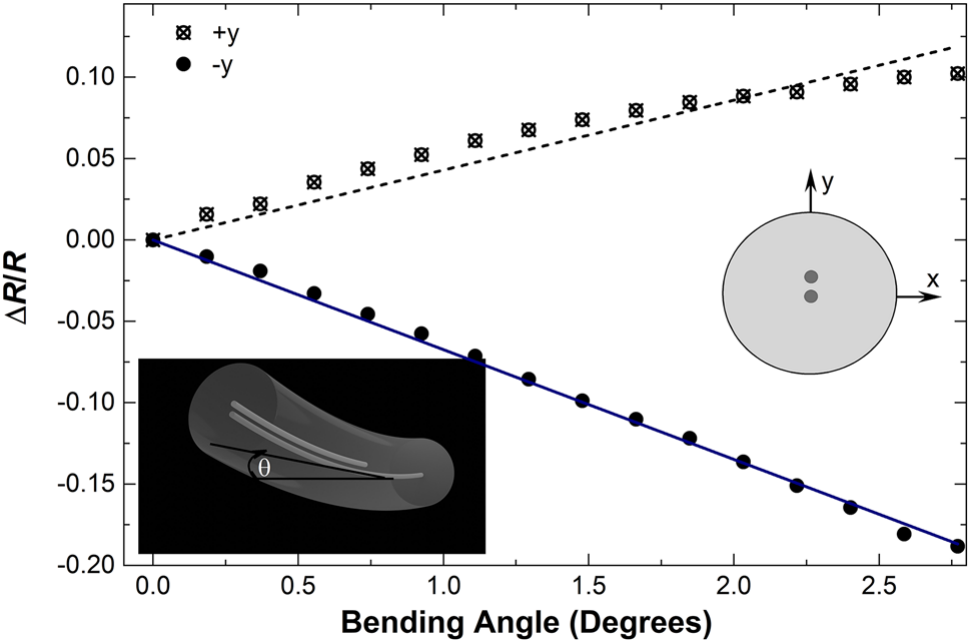
Calibration curves observed when the TCCF was bent in the + *y* and − *y* directions indicated in the drawing. *R* was the reflectivity of the grating when the TCCF was straight and Δ*R* refers to relative reflectivity changes observed at different bending angles. The latter are indicated with the letter θ in the bottom inset figure.

Some observed reflection spectra when the TCCF was bent in the − *y* direction are shown in Fig. 4. The wavelength position of the Bragg grating did not change, however, the reflectivity of the grating decreased in proportion to the bending angle. When the TCCF was bent in the + *y* the opposite was observed, i.e., the reflectivity increased. The calibration curves of the bending in the + *y* and − *y* directions are shown in Fig. 5. In such a figure, Δ*R* = (*R*_*a*_ − *R*_0_), where *R*_a_ was the absolute maximum of the reflection when the bending angle was *a. R*_0_ was the maximum of the reflection when the TCCF was straight.

The bending sensitivities in the + *y* and − *y* directions were found to be, respectively, 0.043 and − 0.067 a.u. per degree. The difference in the sensitivities may be due to the asymmetry of the TCCF. It is worth noting that in both bending directions, Δ*R*/*R* changed linearly with the bending angle and its sign can indicate the direction of the bending.

**Figure 6:**
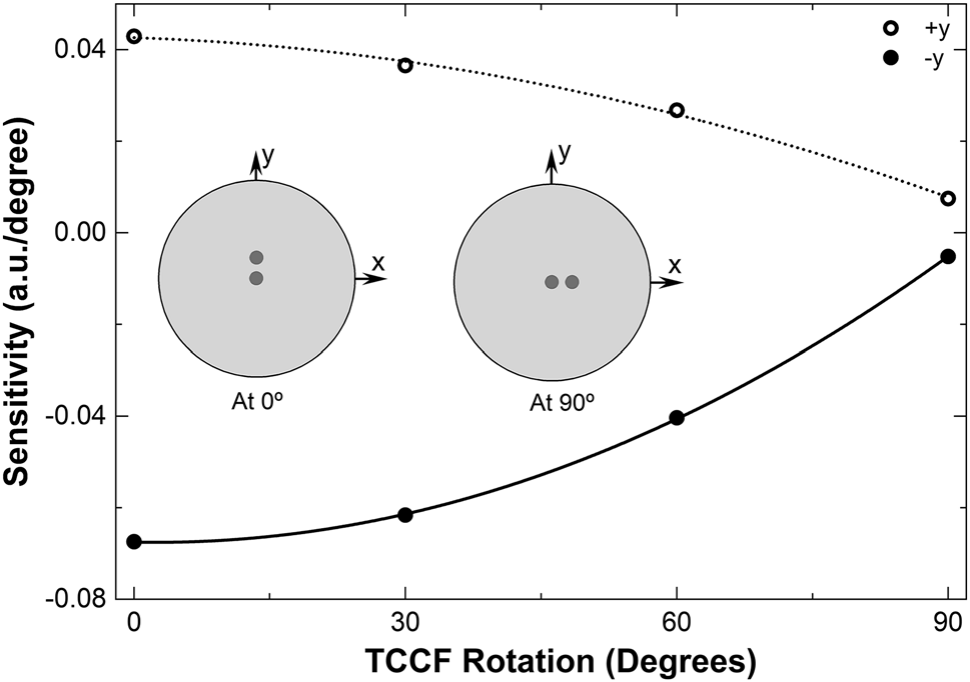
Bending sensitivities measured at different orientations of the TCCF cores with respect to the direction of the bending which was in the + *y* and − *y* directions in all cases. Two orientations of the TCCF are shown in the graph. The rotation angle of the TCCF is measured from *y* to *x* according to the inset illustrations.

To investigate the bending response of our device at different orientations of the fiber cores with respect to the direction of the bending, the TCCF was rotated in the clockwise direction in steps of 30°, at each rotation angle, the fiber was bent in the + *y* and − *y* directions with the same procedure described in the above paragraphs. The bending sensitivities that were found are shown in Fig. 6. The results of such a figure indicate that the bending sensitivity decreases gradually with the orientation of the off-center core of the fiber with respect to the direction of the bending. Thus, if maximum sensitivity is important, the off-center core must be parallel to the direction of the bending.

In an ideal case, the TCCF should be insensitivity to bending when the off-center core is perpendicular to the direction of bending, see the drawing labeled with 90° in Fig. 6. However, due to unavoidable experimental errors and possible imperfections of the TCCF, a low bending sensitivity was observed at such conditions.

The behavior of the TCCF with Bragg gratings can be explained with the results shown in Figs. 2, 3 and 4. The intensity of the light that reaches the two Bragg gratings changes with bending, thus, the light reflected and measured at the central core of the TCCF with a conventional SMF depends on the bending angle. It is important to note from Fig. 5 that a bending angle of 0.2° can be detected. This may be due to the high bending sensitivity of the supermodes excited in the TCCF.

**Figure 7:**
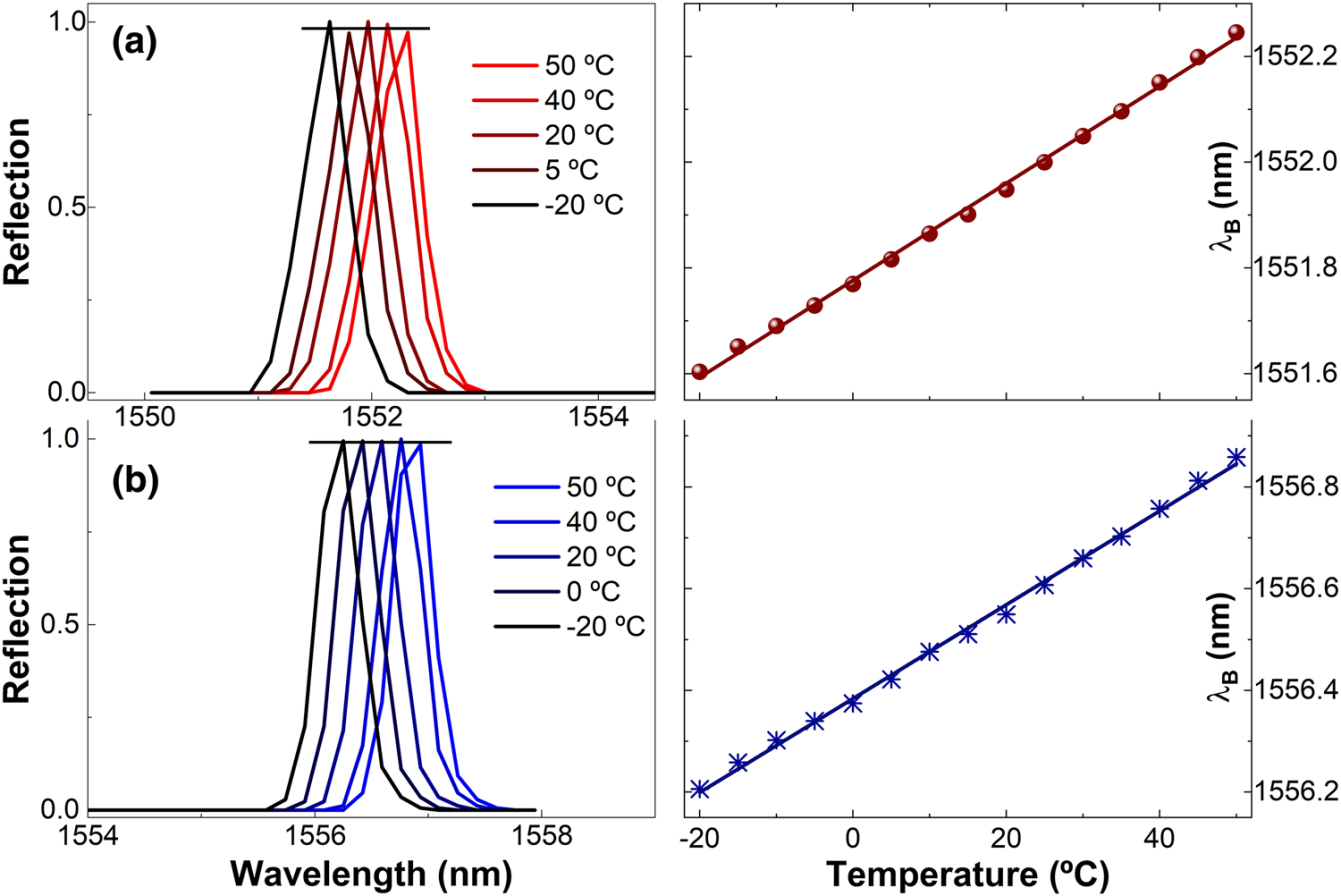
Normalized reflection spectra of coupled-core fiber gratings with Bragg wavelengths around 1552.5 nm (**a**) and 1556.5 nm (**b**) at temperatures indicated in the graphs. The horizontal lines show the fluctuations of the reflectivity of the gratings. The right-hand side graphs show the position of the Bragg wavelengths (dots or asterisks) at different temperatures. The solid lines are a fitting to the experimental data.

The temperature effect on two gratings, one with Bragg wavelength close to 1552 nm and the other close to 1556 nm, was studied from − 20 to + 50 °C. The results of our experiments are shown in Fig. 7. We observed that the relative reflection of the gratings changed little with temperature. From Fig. 7b, the fluctuations of the reflectivity of the grating caused by temperature were found to be around 1.3%. We believe that the low number of data points (512 in 85 nm) of the spectrometer (IMON-512, Ibsen Photonics) that was used to monitor the reflection spectra from the gratings contributed to such fluctuations. The referred spectrometer provided the position of the Bragg wavelength with good accuracy, see the right-hand side graphs of Fig. 7. The temperature sensitivity of the two gratings was found to be ~ 9.30 pm/°C, which is similar to that of a Bragg grating written in conventional single-core optical fibers.

The experimental results presented and discussed above suggest that with our method to fabricate and interrogate coupled-core fibers Bragg gratings, inexpensive intensity-modulated bending sensors that have the capability of distinguishing the direction of the bending can be developed. In this application, the only requirement is to orient the off-center core of the fiber parallel to the direction of the bending. Although in a practical application, temperature compensation may be necessary, but this is a common practice in FBG sensors.

As the gratings here reported were inscribed with UV laser light, they cannot operate at temperatures higher than 300 °C. For such temperatures, gratings inscribed with femtosecond lasers may be an option.

Our bending sensor can be substantially simpler than other FBG bending sensors that need two or more Bragg gratings and expensive interrogation to discriminate the direction of the bending and to compensate the effect of temperature, see for example^25–27,33–35^. Vector bending sensors with Bragg gratings in single-core optical fibers^26,36,37^ or in multicore fibers^27,33,34,38^ have been demonstrated but they are more complex due to their fabrication or interrogation. Other alternatives for sensing bending include the use of coupled core optical fibers in interferometric configurations^32,39–42^, but they also need an expensive wavelength-tracking interrogation method and temperature compensation. Intensity-modulated curvature (bend) sensors based on Bragg gratings written in multicore fibers have been demonstrated recently^43^, but such sensors cannot distinguish the bending direction.

**Figure 8:**
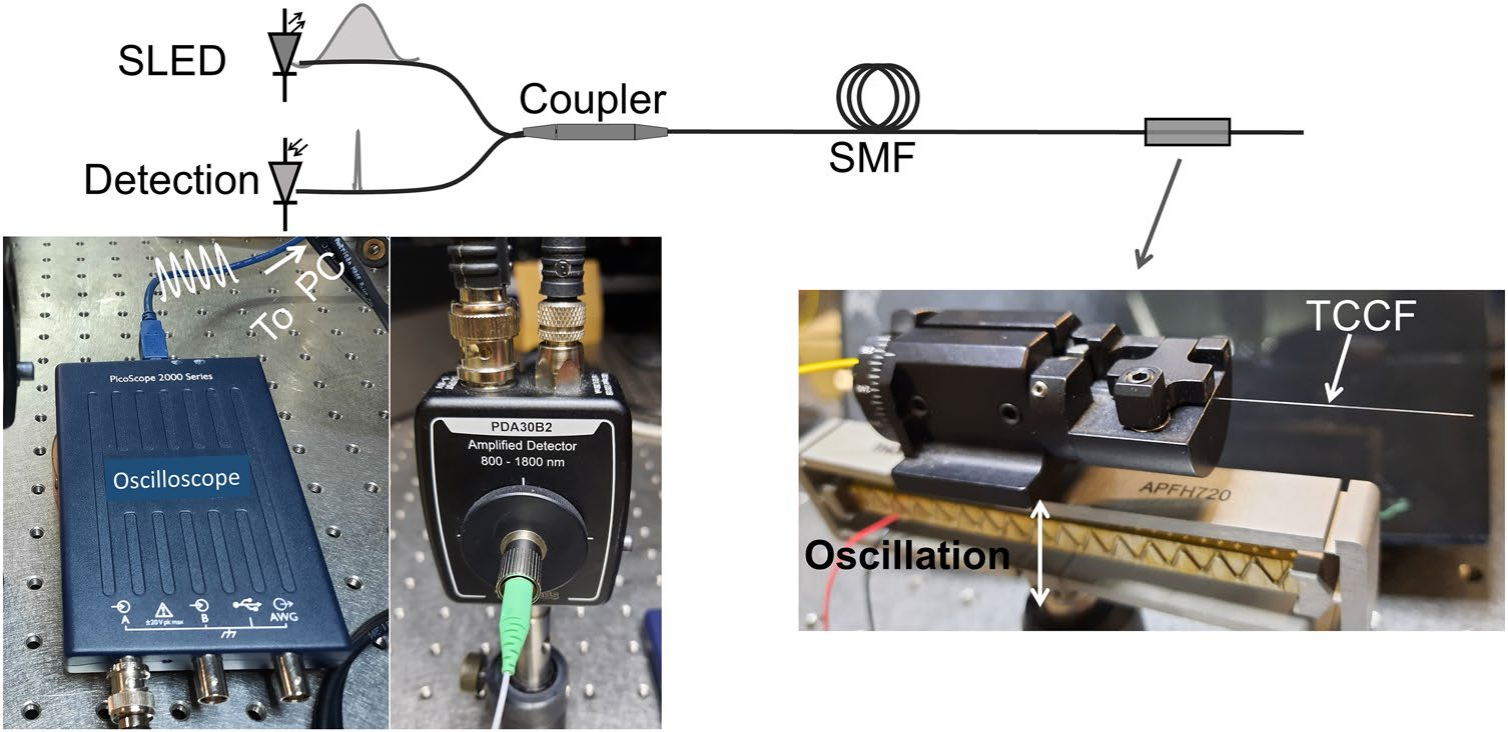
(Top) Schematic of the measuring setup implemented to monitor vibrations. The injected and reflected light from the grating are illustrated with a broad and narrow ‘spectrum’, respectively. (Bottom) Photographs of the mini oscilloscope, photodetector, and piezoelectric actuator with the fiber rotator attached on it. PC is personal computer. The photos were taken by one of the authors (J. Villatoro).

A straightforward application of the devices here proposed is for monitoring vibrations (cyclic bending). To explore this application, the measuring setup shown in Fig. 8 was implemented. TCCF was secured on a fiber rotator and placed in cantilever position with the off-center core oriented in the direction of vibration. In this manner, the segment of TCCF was free to oscillate. An amplified piezoelectric actuator with flexure mount (APFH720, Thorlabs), with maximum displacement of 2500 µm connected to a function generator was used to induce vibrations to the TCCF at different frequencies. To measure reflectivity changes of the coupled-core Bragg grating, a single germanium amplified photodetector (Thorlabs, model PDA30B2) connected to a miniature USB-powered PC oscilloscope (Picoscope, series 2000) was used. The refereed photodetector and oscilloscope are inexpensive. Thus, the detection system of the gratings reported here may cost less than 500 Euros; this means, 10 times lower than the cost of a high-resolution spectrometer that operates around 1550 nm.

Vibrations induced to the TCCF segment were detected as periodic voltage changes with the cited oscilloscope. From such voltage oscillations, we calculated the fast Fourier transform (FFT) with the software provided by the manufacturer of the oscilloscope. We investigated the behavior of the TCCF with gratings at different vibration frequencies.

**Figure 9:**
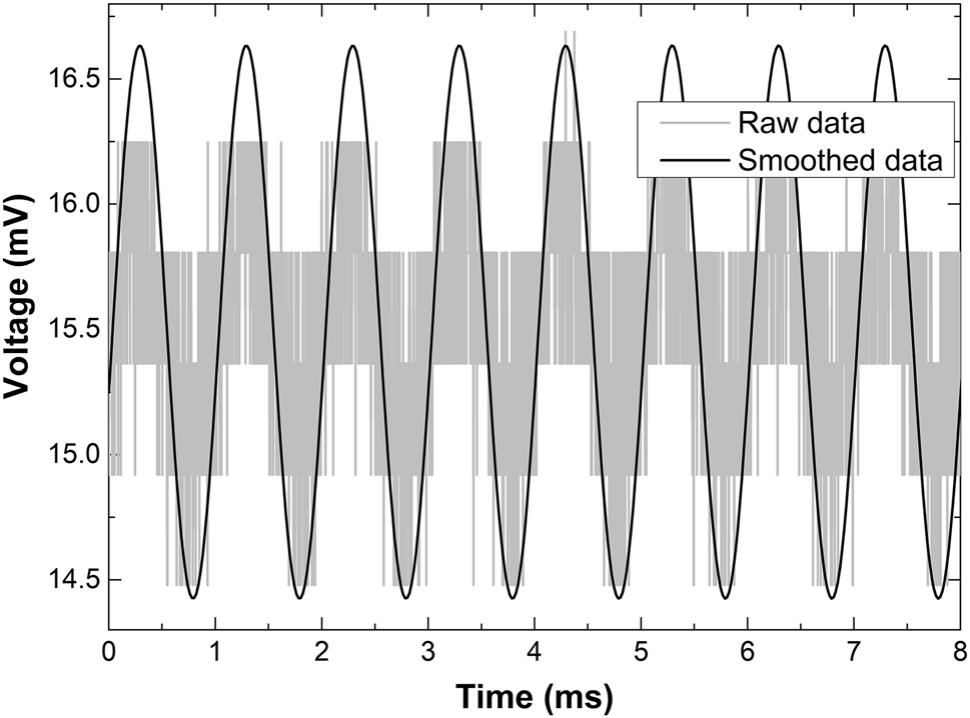
Measured voltage as a function of time when the TCCF was oscillating at 1 kHz.

**Figure 10:**
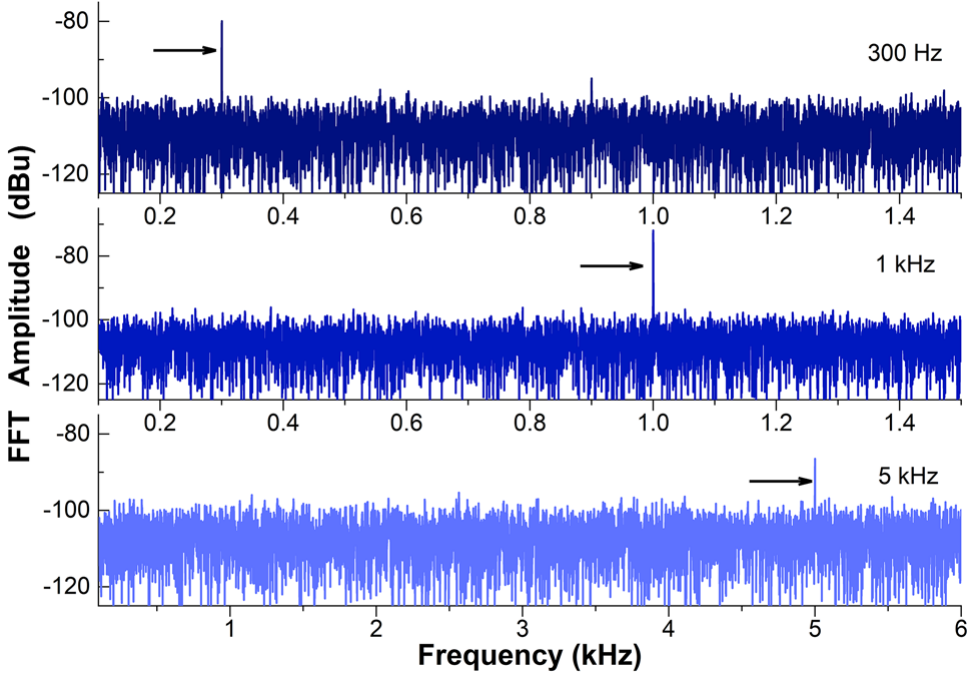
Measured FFT amplitudes as a function of frequency when the TCCF was oscillating at 0.3, 1.0 and 5.0 kHz. A dominant peak is observed at such frequencies.

Figure 9 displays the measured voltage as a function of time when the TCCF with a grating with Bragg wavelength at 1552.5 nm was oscillating at 1 kHz. In the figure, we show the raw and the smoothed data. From the raw data the FFT was calculated. Figure 10 displays the observed FFTs when the TCCF was oscillating at 0.3, 1, and 5 kHz. A dominant peak (indicated with an arrow) located at the input vibration can be observed.

**Figure 11:**
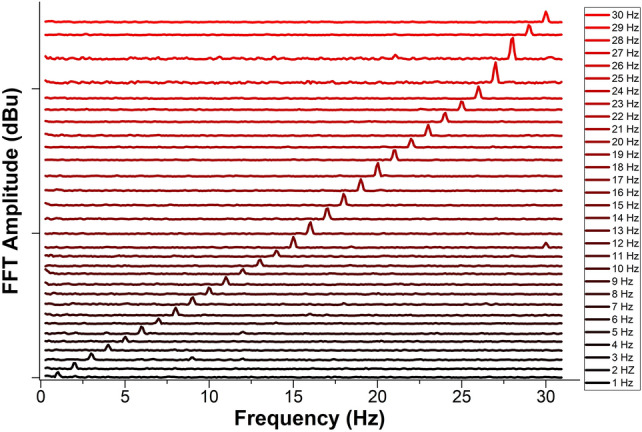
Measured FFT amplitude as a function of frequency. The vertical table shows the input frequencies. A grating with Bragg wavelength of 1552.5 nm was used.

We also investigated the capability of our device to measure lower frequencies. Figure 11 displays the measured FFTs when the TCCF was vibrating at frequencies between 1 and 30 Hz. Again, a dominant peak can be seen at each input frequency.

It is worth noting that the device responded well for low and high frequencies. Thus, it can be useful to monitor events like seism, ground motions, or small movements of critical infrastructures or machines. For such applications, our vibration sensor is substantially simpler than several FBG-accelerometers reported in the literature, see for example^2^, which monitor shifts of the Bragg wavelength. However, for practical applications, our coupled-core fiber Bragg gratings must be properly packaged as bare optical fibers can be easily broken when they are bent.

## Conclusions

In this work, we have demonstrated that Bragg gratings inscribed in an optical fiber with two identical coupled cores can be used to develop intensity-modulated sensors. We have demonstrated that the reflectivity of the Bragg gratings changed in proportion to the bending applied to the coupled-core fiber. Thus, the reflection changes from the Bragg grating can be monitored easily with a low-cost photodetector. An important advantage is that temperature alters little the reflectivity of the Bragg grating but it causes changes in its wavelength position.

The results reported here could be useful in the design of a variety of inexpensive sensors based on coupled-core fiber with Bragg gratings for practical applications. Intensity-modulated bending and vibrations sensing were demonstrated. The advantage of our sensors include capability of distinguishing the bending direction or detecting vibrations in a broad range. We demonstrated the detection of frequencies from 1 Hz to 5 kHz. Any other parameter that can be converted in local or periodic bending to the coupled-core fiber, hence, in point or periodic intensity changes can also be detected. Thus, it seems feasible to develop sensors to monitor pressure, impact, force, touch, etc.

Sensors for monitoring fast phenomena, as for example impacts, ultrasound, etc., may be developed with coupled-core fiber with Bragg gratings because intensity changes can be monitored at high speed, up to MHz. Multiplexing of coupled-core fiber Bragg grating sensors seems also feasible. For example, with a 1 × N coupler, it would be possible to interrogate N sensors. Although, wavelength division multiplexers and demultiplexers, which are commercially available, may be necessary to discriminate reflection changes of several sensors with different Bragg wavelengths.

We believe that the concepts and results shown in this work may expand the applications of Bragg gratings in the sensing field and perhaps in other applications. They also may open up new lines of research that lead to novel and functional sensors that are less expensive than their counterparts based on Bragg gratings written in conventional single-core optical fibers.

## Data Availability

Data underlying the results presented in this paper are not publicly available at this time but may be obtained from the authors upon reasonable request.
